# Regulatory Network of the Scoliosis-Associated Genes Establishes Rostrocaudal Patterning of Somites in Zebrafish

**DOI:** 10.1016/j.isci.2019.01.021

**Published:** 2019-01-21

**Authors:** Sevdenur Keskin, M. Fethullah Simsek, Ha T. Vu, Carlton Yang, Stephen H. Devoto, Ahmet Ay, Ertuğrul M. Özbudak

**Affiliations:** 1 Division of Developmental Biology, Cincinnati Children's Hospital Medical Center, Cincinnati, OH 45229, USA; 2 Department of Pediatrics, University of Cincinnati College of Medicine, Cincinnati, OH 45229, USA; 3 Department of Genetics, Albert Einstein College of Medicine, Bronx, NY 10461, USA; 4 Departments of Biology and Mathematics, Colgate University, Hamilton, NY 13346, USA; 5 Department of Biology, Wesleyan University, Middletown, CT 06459, USA

**Keywords:** Biological Sciences, Developmental Biology, Embryology, Mathematical Biosciences

## Abstract

Gene regulatory networks govern pattern formation and differentiation during embryonic development. Segmentation of somites, precursors of the vertebral column among other tissues, is jointly controlled by temporal signals from the segmentation clock and spatial signals from morphogen gradients. To explore how these temporal and spatial signals are integrated, we combined time-controlled genetic perturbation experiments with computational modeling to reconstruct the core segmentation network in zebrafish. We found that Mesp family transcription factors link the temporal information of the segmentation clock with the spatial action of the fibroblast growth factor signaling gradient to establish rostrocaudal (head to tail) polarity of segmented somites. We further showed that cells gradually commit to patterning by the action of different genes at different spatiotemporal positions. Our study provides a blueprint of the zebrafish segmentation network, which includes evolutionarily conserved genes that are associated with the birth defect congenital scoliosis in humans.

## Highlights

• A core network establishes rostrocaudal polarity of segmented somites in zebrafish
• *mesp* genes link the segmentation clock with the FGF signaling gradient
• Gradual patterning is done by the action of different genes at different positions

## Graphical Abstract

**Figure:**
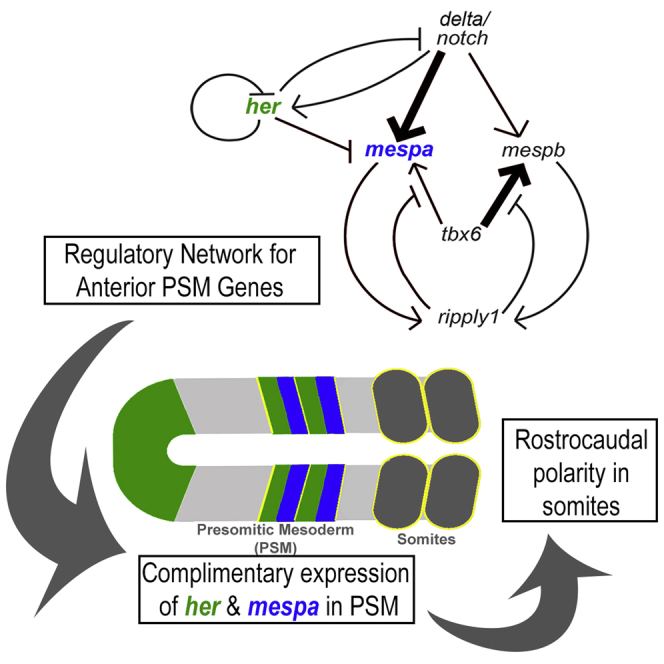

## Introduction

**Figure 1:**
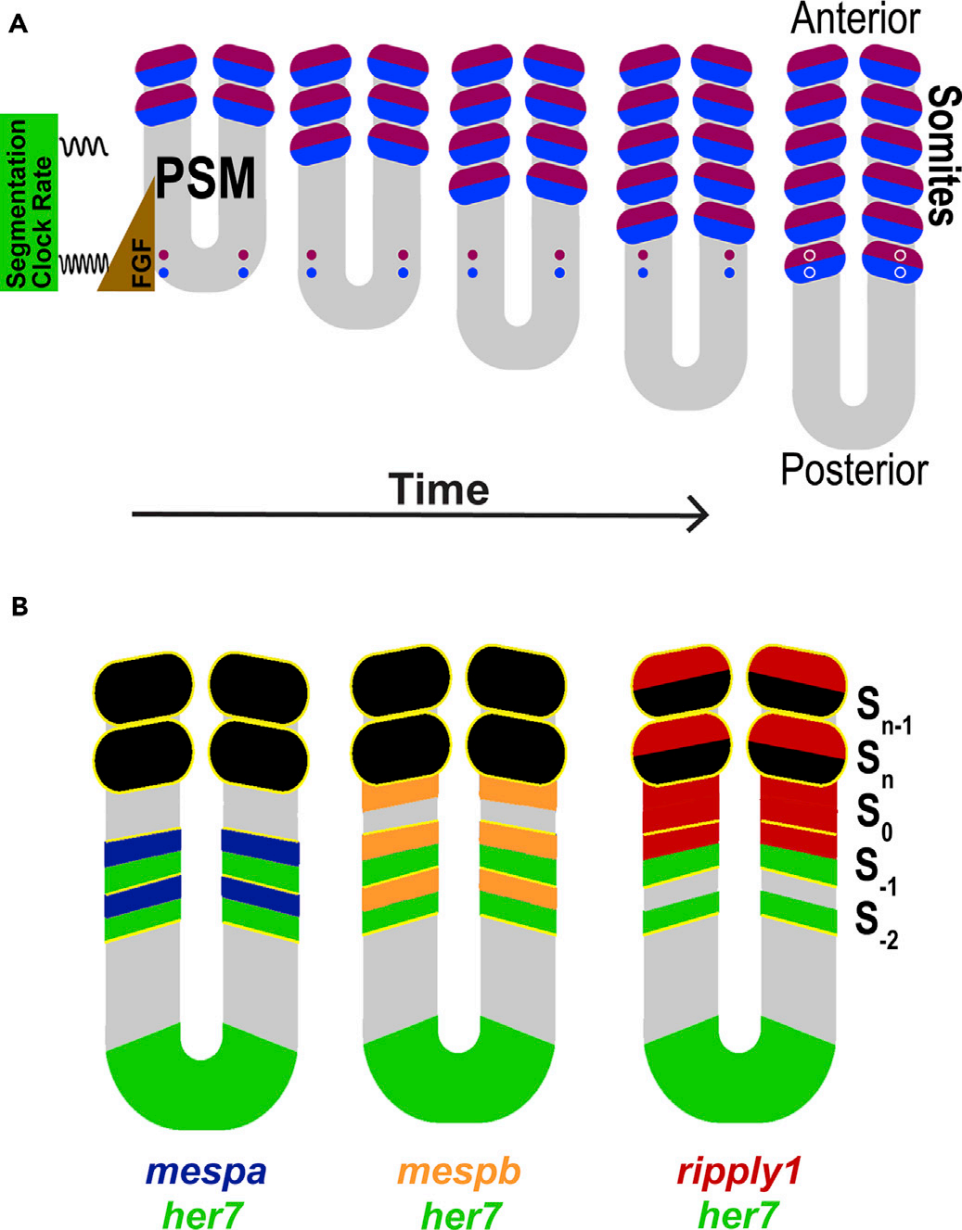
Somite Development (A) Tracing of a pair of cells destined to become somites. Bilateral somite generation is shown with the differences in final gene expression (purple or blue) based on the phase of the segmentation clock when the cell incorporated into a somite and the movement of a pair of cells into a somite. A pair of cells destined to differentiate into two different tissues derived from a somite is shown as a purple circle and a blue circle. The cells ingress into the tail (posterior) end of PSM through processes of gastrulation and germ layer formation. As more cells enter the posterior PSM, the pair gradually moves anteriorly, eventually reaching the anterior-most boundary of the PSM. The pair of cells then becomes incorporated into a somite (oval). During this trajectory, the cells are exposed to a succession of different signals and express sets of genes in a spatiotemporally ordered manner. High FGF (brown) in the posterior region maintains the cells in an undifferentiated and developmentally plastic state. In the posterior region the segmentation clock oscillates rapidly. As the cells move into the anterior region of the PSM, the oscillation rate decreases and other genes begin to be dynamically expressed. As the cells become established in the posterior compartments of the prospective somite segments, the expression of tissue-specific genes become stable. Because different sets of genes are expressed in cells located in complementary (anterior or posterior) compartments of somites, these cells adopt different fates. The final fate of the cell depends on its phase of oscillation as it exits from the anterior end of PSM. This model is based on Pourquie (2011). (B) Diagram of the expression domains of clock genes (green), *mespaa/mespab* (dark blue), *mespba/mespbb* (orange), and *ripply1* genes (dark red). Yellow lines show boundaries of predetermined and formed somites.

A challenge in developmental biology is providing a molecular description of the cascade of regulatory steps that result in morphological changes and cell differentiation. Vertebrate somite segmentation is an example of a regulatory cascade and provides a system for studying the coordinated expression of multiple genes controlled by interconnected signaling pathways. Errors in this regulatory cascade result in various birth defects, including congenital scoliosis (Pourquie, 2011). Somites, the embryonic origin of the body segments of a vertebrate, are produced sequentially from the presomitic mesoderm (PSM) at the tail end of the embryo as the PSM elongates posteriorly (Figure 1A). In the zebrafish, one bilateral pair of somites segments at the anterior end of the PSM every 30 min. The timing of the separation of the somites from the PSM, referred to as somite periodicity, is controlled by a segmentation clock in the posterior PSM. This segmentation clock exhibits oscillatory expression of “clock” genes (Pourquie, 2011). The pacemaker mechanism of the segmentation clock relies on the auto-inhibitory feedback loop of *her* (in zebrafish) or *Hes* (in mouse) gene expression (Ay et al., 2013, Giudicelli et al., 2007, Harima et al., 2013, Jensen et al., 2003, Lewis, 2003, Monk, 2003, Schroter et al., 2012).

The positions of segment boundaries are instructed by the posteroanterior gradient of fibroblast growth factor (FGF) signaling (Simsek and Ozbudak, 2018). The high FGF signal in the posterior region keeps the cell in an undifferentiated and developmentally plastic state in which the segmentation clock “ticks” (expression of clock genes oscillate) at a steady rapid rate (Pourquie, 2011). As the cell shifts into the anterior region of the PSM, the period of gene oscillations increases; consequently, the segmentation clock genes display dynamic expression patterns (stripes) in the PSM (Figure 1B). As the cells shift anteriorly and the oscillation period increases, the stripes of clock gene expression become narrower and become established in the posterior compartments of the prospective somite segments. In parallel, additional genes begin to be expressed dynamically in the anterior PSM. The expression of the genes becomes stable (zero or non-zero amounts) as the cell emerges from the PSM and becomes incorporated into a somite (Figure 1A). Different sets of genes are expressed in cells located in complementary (anterior or posterior) compartments of somites (rostrocaudal polarity), and thereby they govern the consequent differentiation of segmented cells (Holley, 2007, Stellabotte and Devoto, 2007). The final state of the cell depends on its phase of oscillation as it exits from the anterior end of PSM. In this way, the temporal oscillation in the posterior PSM is mapped into a periodic spatial pattern of cells in different states in the formed somites. Although the mechanism that generates the striped pattern of clock gene expression is well studied (Ay et al., 2013, Ay et al., 2014, Harima et al., 2013, Schroter et al., 2012), the regulatory network that establishes rostrocaudal somite polarity is not well characterized. The signaling pathways implicated in the establishment of rostrocaudal somite polarity include the FGF, Wnt, and Notch pathways (Pourquie, 2011).

Mutations of genes in the *HES* and *MESP* families of transcription factors result in scoliosis (Turnpenny et al., 2007), indicating their importance in somite development. *Mesp* genes encode transcription factors of the bHLH family, and *Mesp2* deletion in mouse disrupts segmentation and rostrocaudal polarity of somites (Saga et al., 1997). In zebrafish, four *Mesp2* homologs (*mespaa, mespab, mespba*, and *mespbb*) are dynamically expressed in the anterior PSM, with peaks that coincide with troughs in the expression patterns of the clock genes *her1* and *her7* and the gene *deltaC*, encoding a ligand of the Notch pathway (Cutty et al., 2012, Sawada et al., 2000) (Figure 1B). In the PSM, *mesp* genes are expressed in the rostral compartment, whereas *her* and *deltaC* genes are expressed in the caudal compartment of the next segmenting somite. This complementarity is a prerequisite for the rostrocaudal polarity of segmented somites. Although rostrocaudal polarity is disrupted in zebrafish *mesp* mutants, mutation of the *mesp* family genes results in milder segmentation defects in zebrafish than in mouse owing to genetic and functional redundancy (Yabe et al., 2016). In zebrafish, the transcriptional repressor encoded by *ripply1* is expressed in stripes in the anterior PSM and in recently formed somites (Kawamura et al., 2005). Morpholino-oligonucleotide-mediated knockdown of *ripply1* in zebrafish or knockout of *Ripply2* in mice causes segmentation defects (Kawamura et al., 2005, Morimoto et al., 2007). Mutations in *MESP2* and *RIPPLY2* are present in patients with congenital scoliosis (Cornier et al., 2008, Turnpenny et al., 2007) or Klippel-Feil syndrome (Karaca et al., 2015), respectively. Despite their developmental and medical importance, the regulatory relationships between the segmentation clock and the *Mesp* and *Ripply* genes are not completely understood. Joint knockdown, using morpholino-oligonucleotides, of *her*1 and *her7* results in loss of rostrocaudally polarized expression of *mesp* genes in zebrafish (Henry et al., 2002, Oates and Ho, 2002). Whether this effect on the *mesp* gene expression pattern is due to a rapid action by Her family transcription factors or indirectly through Notch signaling is not clear.

To assess the regulatory relationships between the segmentation clock and morphogen signaling cascades in somite patterning, we have studied somite segmentation in zebrafish by altering the expression of specific genes in a time-controlled manner and by assessing the impact on the expression of other genes at defined times during development (Lewis and Ozbudak, 2007). Because cells at various stages of somitogenesis are distinctly positioned along the anteroposterior axis, global perturbation of gene expression abruptly at specific time during development will result in cells in different positions along the body axis experiencing the disturbance at different times relative to the time of their exit from the PSM. Thus the resulting somite segmentation pattern provides a map of the effects of the disturbance of gene expression relative to the time of exit from the PSM (Lewis and Ozbudak, 2007).

Here, we constructed a regulatory network that incorporates the zebrafish homologs of scoliosis-linked genes and provides a mechanism for the establishment of rostrocaudal segment polarity. We combined time-controlled perturbation experiments at high temporal resolution in zebrafish embryos with mathematical modeling to determine that rostrocaudally polarized expression of genes encoding the Mesp family of transcription factors is established by both the temporal action of the segmentation clock and the spatial action of FGF signaling gradient. We also showed that cells are gradually time stamped: their eventual state in the somite is dictated by the action of different transcription factors sequentially at different spatiotemporal positions during segmentation. Computational simulations of the segmentation network recapitulated the complementary expression of the families of *her* and *mesp* genes in wild-type embryos and the effects of perturbation experiments at the time window of 30 min to 4 h. This study provides a blueprint of the zebrafish segmentation network, incorporating genes with homologs in humans that are associated with congenital scoliosis.

## Results

### Transcription of *mespaa* Is Rapidly Repressed by the Segmentation Clock

**Figure 2:**
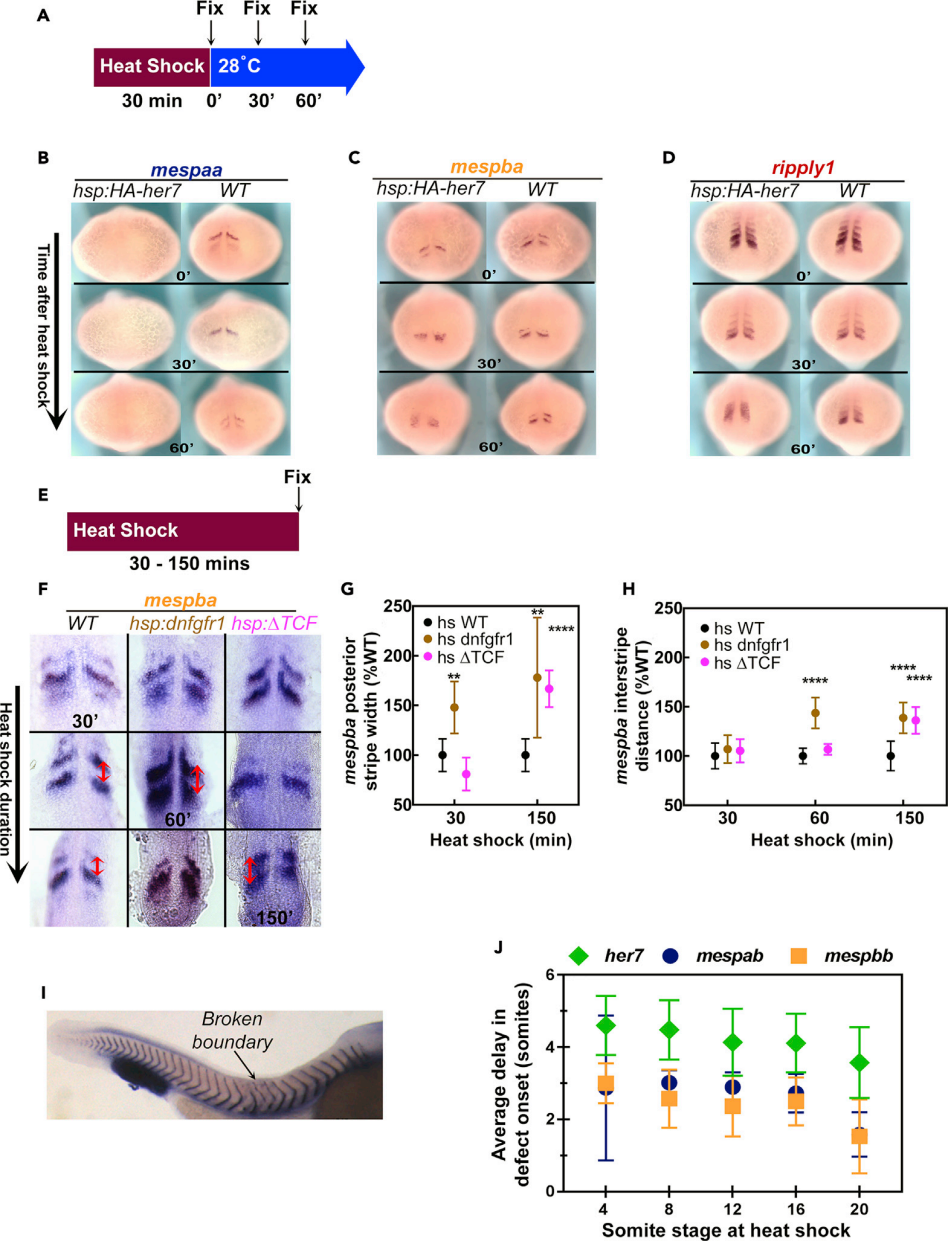
Expression of *mesp* Genes Read Out the Segmentation Clock Genes and FGF Signaling (A) Embryos from different genetic backgrounds were fixed immediately or after 30 min, or 60 min of recovery following 30-min heat shock at 37°C. (B–D) ISH images of *mespaa* (B)*, mespba* (C), and *ripply1* (D) after heat shock of *tg(hsp70l:HA-her7)* and wild-type (WT) embryos at different recovery time points. This experiment was repeated twice and 43–49 embryos analyzed for all probes and time points. (E) Embryos from different genetic backgrounds were fixed immediately after 30, 60, 90, 120, or 150 min of heat shock at 37°C. (F) Flat mounted ISH images of *mespba* after heat shock of *tg(hsp70l:dnfgfr1a-EGFP)*, *tg(hsp70l:tcf7l1a-GFP),* and wild-type (WT) embryos at different time points. For each time point 8 to 22 embryos were analyzed. Red arrows show the interstripe distance, which was measured between the anterior ends of stripes. (G and H) Effect of inhibition of FGF (brown) or Wnt (pink) signaling on the width of the posteriormost stripe of *mespba* expression stripe (G) and the interstripe distance between two stripes of *mespba* expression (H) shown as percent of that in wild-type embryos exposed to heat shock. Error bars indicate one standard deviation. (I) ISH image of *xirp2* showing position of the somite boundary defects in a *tg(hsp70l:HA-her7)* embryo after 40 min of heat shock. Heat shock started when embryo was at 4-somite stage; first broken boundary appears between 9^th^ and 10^th^ somites as indicated. (J) Average delay in onset of segmentation defects in embryos overexpressing *her7* [*tg*(*hsp70l:HA-her7*)]*, mespab* [*tg*(*hsp70l:mespab-myc*)], or *mespbb* [*tg*(*hsp70l:mespbb-myc*)] at different somite stages after heat shock at 37°C. *her7* transgenic embryos were subjected to heat shock for 40 min; *mespab* and *mespbb* transgenic embryos were subjected to heat shock for 30 min. Experiments were repeated twice, and 30–62 embryos were analyzed for all genotypes and stages. Error bars indicate one standard deviation. See also Figure S1.

Owing to the complementary expression of *her* and *mesp* family genes, we hypothesized a regulatory interaction between them. To determine if *mesp* genes are regulated by segmentation clock transcription factors, we performed time-controlled perturbation experiments. We used the *hsp70l:HA-her7* transgenic line to overexpress the *her7* clock gene by heat shock. We compared the expression pattern of *mespaa*, *mespba*, and *ripply1* in wild-type embryos and the *hsp70l:HA-her7* transgenic embryos immediately after a 30-min heat shock and 30 or 60 min after the 30-min heat shock (Figure 2A). *In situ* hybridization (ISH) experiments in the embryos overexpressing *her7* revealed reduced amounts of *mespaa* transcripts with their mRNAs barely detectable or undetectable immediately after the 30-min heat shock (Figure 2B). In contrast, *mespba* and *ripply1* showed reduced and altered patterns of expression by 30 min after the heat shock period. Cells expressing high and low levels of *mespba* or *ripply1* were intermingled by 60 min after the heat shock period (Figures 2C and 2D), reflecting desynchronization of expression patterns among neighboring cells (Ozbudak and Lewis, 2008). The reduction in *mespaa* in the *hsp70l:HA-her7* transgenic lines within the 30-min heat shock treatment suggested a potential direct regulation of *mespaa* by Her7, whereas the delay in the change in *mespba* and *ripply1* transcripts suggested an indirect regulation of *mespba* and *ripply1* by Her7 (Figures 2B–2D).

### *mesp* Genes Respond to the FGF Signaling Gradient

The position after which cells become nonresponsive to perturbations in the FGF signaling gradient is called the site of segmental determination or determination front (Dubrulle et al., 2001, Sawada et al., 2001, Simsek and Ozbudak, 2018). Perturbation of FGF or Wnt signaling shifts the spatial onset of expression of *mesp* genes (Aulehla et al., 2008, Bajard et al., 2014, Delfini et al., 2005, Sawada et al., 2001). To elucidate which signal defines the spatial domain of *mesp* expression, we performed time-controlled perturbation experiments. As we have done previously (Simsek and Ozbudak, 2018), we used heat-shock-inducible transgenic lines to inhibit the activities of each signaling pathway in a time-controlled manner. We used *hsp70l:dnfgfr1a-EGFP*, expressing dominant-negative FGF receptor fused with green fluorescent protein (GFP) reporter, to block FGF signaling (Lee et al., 2005) and *hsp70l:tcf7l1a-GFP*, expressing dominant-negative *tcf7l1a* fused with GFP reporter, to block Wnt-regulated transcriptional responses (Lewis et al., 2004). We subjected transgenic or wild-type embryos to heat shock for various durations (30–150 min) and fixed the embryos right after heat shock (Figure 2E). We performed ISH against *mespaa* (Figure S1) and *mespba* transcripts (Figure 2F) and measured the distance between “stripes” of *mespba* expression and the width of the posterior *mespba* stripe (Figures 2G and 2H). We observed an immediate increase in the width of the posteriormost *mespaa* or *mespba* stripes after the 30-min inhibition of FGF signaling. We also observed an increase in the interstripe distance, resulting from a posterior shift in the expression stripe, in the next segmentation cycle (60 min heat shock). In contrast, inhibition of Wnt signaling increased the width of the *mespaa* and *mespba* stripes only after a long delay after heat shock (Figures 2F–2H and S1). These data suggested a more direct effect of FGF signaling on *mesp* expression and an indirect effect of Wnt signaling. The striped pattern of expression is consistent with *mesp* genes responding to the temporal information from the clock with FGF providing spatial regulation of expression.

### Different Transcription Factors Function at Different Positions to Control Segmentation

*Mesp* genes are expressed in only the anterior portions of prospective somites (Cutty et al., 2012, Morimoto et al., 2005, Sawada et al., 2000). Studies in mice indicated that the juxtaposition of cells expressing *Mesp* genes with those not expressing them (rostrocaudally polarized expression) is a requirement for the formation of the segment boundary in the last-forming somite (Morimoto et al., 2005). Mutations of *mesp* family genes result in segmentation defects in zebrafish that are less severe than those that result in mice. This phenotypic difference is attributed to both genetic redundancy (presence of duplicated *mesp* genes in zebrafish) and functional redundancy (action of yet-to-be discovered genes functionally equivalent to *mesp* genes) (Yabe et al., 2016). We investigated the role of rostrocaudally polarized expression of *mesp* genes for segment boundary formation in zebrafish using transgenic lines (Windner et al., 2015) to overexpress *mespab* and *mespbb* in a time-controlled manner during somitogenesis.

Heat-shock-driven expression of *mespab* or *mespbb* for 30 min or *her7* for 40 min did not disrupt the next 1 to 3 somites that formed but resulted in defects in somites that formed later (Figures 2I, and 2J). We monitored somite boundaries by ISH for *xirp2* (Figure 2I). For example, we observed a defect in somite boundary establishment between the 9^th^ and 10^th^ somites in an embryo subjected to heat-shock-mediated induction of *her7* at the 4-somite stage (Figure 2I). The delay in the onset of the boundary defect decreased as the genes were induced later during somitogenesis (Figure 2J). These results showed that rostrocaudally polarized expression of *mesp* genes is not required for the next forming somite but is required for somites that form more posteriorly. Because segmentation defects started later in *her7* overexpression than *mespab* or *mespbb* overexpression at all stages (Figure 2J), these results suggested that rostrocaudally polarized expression of *mesp* genes is necessary at a later time point (at more anterior positions) than the action of the segmentation clock (Figures 2I and 2J). Collectively, these results suggested that *mesp* genes function in the transfer of temporal information from the segmentation clock to the spatial patterning of somite segments. Furthermore, genes in the *her* and *mesp* families function at different times and positions after which cells no longer need their action to execute segmental pattern formation.

### Notch Signaling Activates Rostrocaudally Expressed *mesp* and *her* Genes with Different Kinetics

Based on mutant and transgenic phenotypes in mouse, it was proposed that Notch signaling activated by the ligand Delta is an output of the segmentation clock, and Notch signaling drives striped expression of *Mesp2* in the anterior PSM and thereby establishes anteroposterior compartmentalization of somites (Oginuma et al., 2010). According to this model, overactivation or inhibition of Notch signaling should disturb rostrocaudally polarized expression of *Mesp* genes and rapidly produce segmentation defects. This model is inconsistent with the following published data: (1) zebrafish and mouse embryos with hyperactive Notch signaling throughout the PSM still form more than 10 somites (Feller et al., 2008, Ozbudak and Lewis, 2008) and (2) segmentation proceeds for many cycles when Notch signaling is inhibited in both zebrafish and mouse (Huppert et al., 2005, Mara et al., 2007, Ozbudak and Lewis, 2008, Pourquie, 2011, Riedel-Kruse et al., 2007).

**Figure 3:**
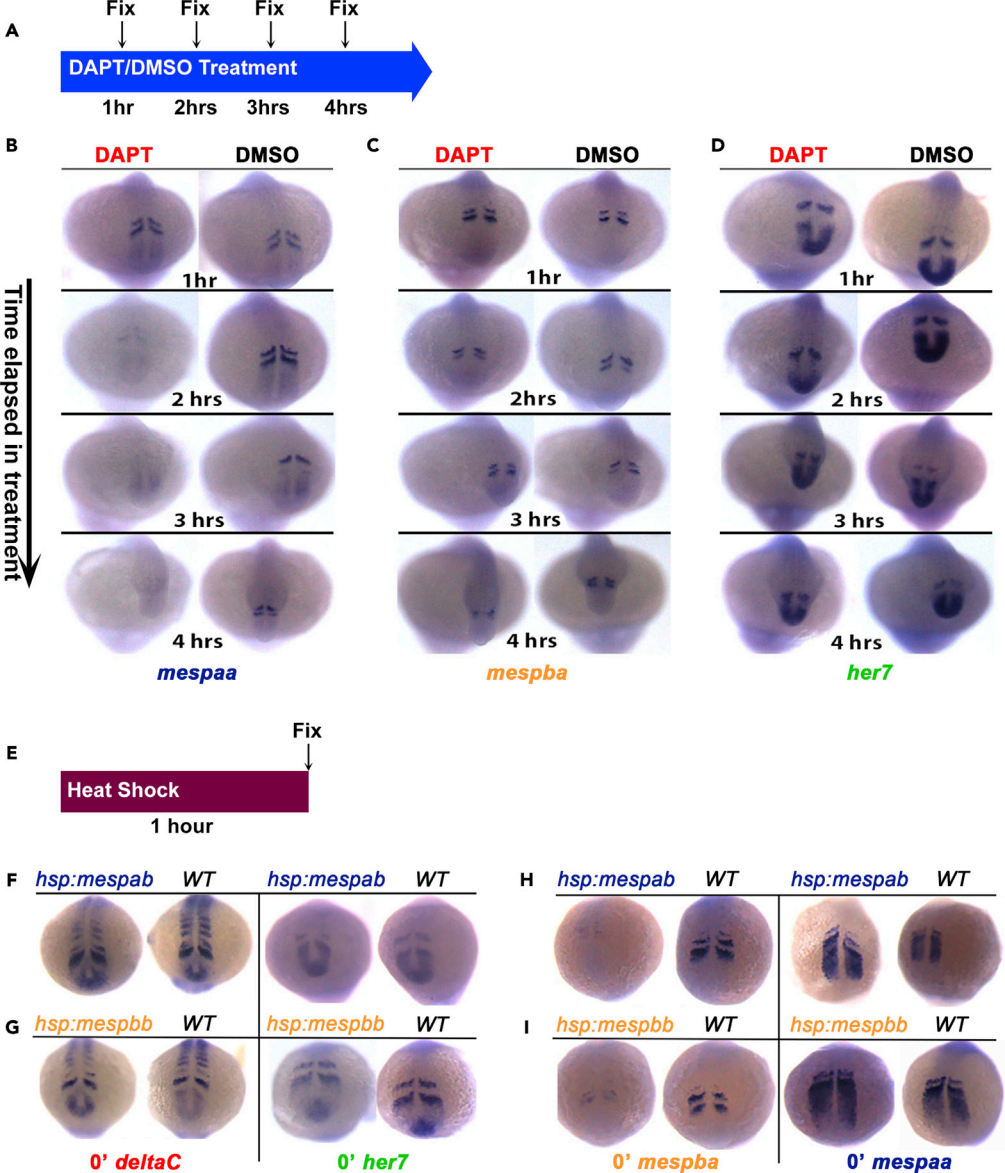
Gene Regulatory Network in the Anterior PSM Is Mapped (A) Wild-type (WT) embryos were continuously treated in DAPT or DMSO and fixed at different time points. (B–D) ISH pictures for (B) *mespaa,* (C) *mespba*, and (D) *her7* genes in DAPT- or DMSO-treated WT embryos. These experiments were repeated twice, and 38–52 embryos were analyzed for all conditions, probes, and stages. (E) Embryos from different genetic backgrounds were fixed immediately after 60 min of heat shock at 37°C. (F and G) ISH images of the expression pattern of *her7* and *deltaC* immediately after 1-h heat shock in (F) *hsp70l:mespab-myc* or (G) *hsp70l:mespbb-myc* embryos. (H and I) ISH images of *mespba* or *mespaa* expression immediately after 1-h heat-shock in (H) *hsp70l:mespab-myc* or (I) *hsp70l:mespbb-myc* embryos. (F–I) These experiments were repeated twice, and 50–65 embryos were evaluated for all genotypes, probes, and stages.

To clarify the role of Delta/Notch signaling, we blocked this pathway by treating embryos with γ-secretase inhibitor,N-[N-(3,5- difluorophenacetyl)-L-alanyl]-S-phenylgly cine t-butyl ester (DAPT) during somitogenesis in a time-controlled manner (Figure 3A). The results showed that expression of *mespaa*, but not *mespba* or *her7*, became barely detectable within 2 h of inhibition of Notch signaling (Figures 3B–3D). Consistent with previous work (Ozbudak and Lewis, 2008), the pattern of *her7* expression was disrupted after 4 h (Figure 3D). Disruption in *mespba* expression occurred after 4 h (Figure 3C). These results suggested that expression of *mespaa* had a stronger dependence on Notch signaling than does the expression of *mespba* and *her7*, because *mespba* and *her7* expression became gradually desynchronized over a prolonged time period when Notch signaling is blocked and *mespaa* expression was affected more quickly. Redundancy in the *mesp* genes and the relatively low effect that we observed on *mespba* compared with *mespaa* in response to loss of Notch signaling may explain why segmentation proceeds normally over a long time when Notch signaling is disrupted. Therefore our data support a model in which Notch signaling primarily functions upstream of the segmentation clock in the posterior end of PSM and not downstream of the segmentation clock in the anterior PSM, as was proposed previously (Oginuma et al., 2010).

### Scoliosis-Linked Genes Are Connected in a Regulatory Network

Mutations in *Delta*, *Mesp*, and *Hes* genes result in scoliosis in patients (Giampietro et al., 2009, Pourquie, 2011), and mutations of their orthologous genes in the mouse (zebrafish) model completely (partially) recapitulate the phenotype (Lleras Forero et al., 2018, Wopat et al., 2018). Although the functional importance of these genes has been better established in the mouse model (Morimoto et al., 2005, Oginuma et al., 2010), the dynamic regulatory relationships among these genes have not been determined in any model organism. To identify the dynamic regulatory relationships among *delta*, *mesp*, and *hes* genes, we overexpressed *mespab* and *mespbb* genes in a time-controlled manner and assessed their impact on the expression of *her7, deltaC, mespaa*, and *mespba* by performing ISH at different time intervals. For these experiments, we used a 60-min heat shock (Figure 3E). Expression of *her7* was not affected by overexpression of either *mesp* genes, suggesting that there is no feedback loop between the clock and *mesp* genes (Figures 3F and 3G). Expression of *deltaC* was also not affected by overexpression of either *mesp* genes, suggesting that there is no feedback loop between the Delta/Notch signaling and *mesp* genes (Figures 3F and 3G). The genes *mespaa* and *mespab* are paralogs, as are *mespba* and *mespbb*. We used transgenic lines to overexpress one paralog and ISH probes to detect the changes in the expression levels of the other paralog gene. Overexpression of either of the *mespab* or *mespbb* genes reduced the expression of *mespba,* but not of *mespaa* (Figures 3H and 3I). However, transcription of *mespba* is reduced with 60-min heat shock (Figures 3H and 3I), but not 30-min heat shock (data not shown), suggesting that these regulations are indirect. These results are consistent with (and extend) the previous finding that transcription of *mespb* is reduced by an indirect negative feedback loop from Mespb that includes Ripply1 and Tbx6 (Takahashi et al., 2010, Windner et al., 2015).

**Figure 4:**
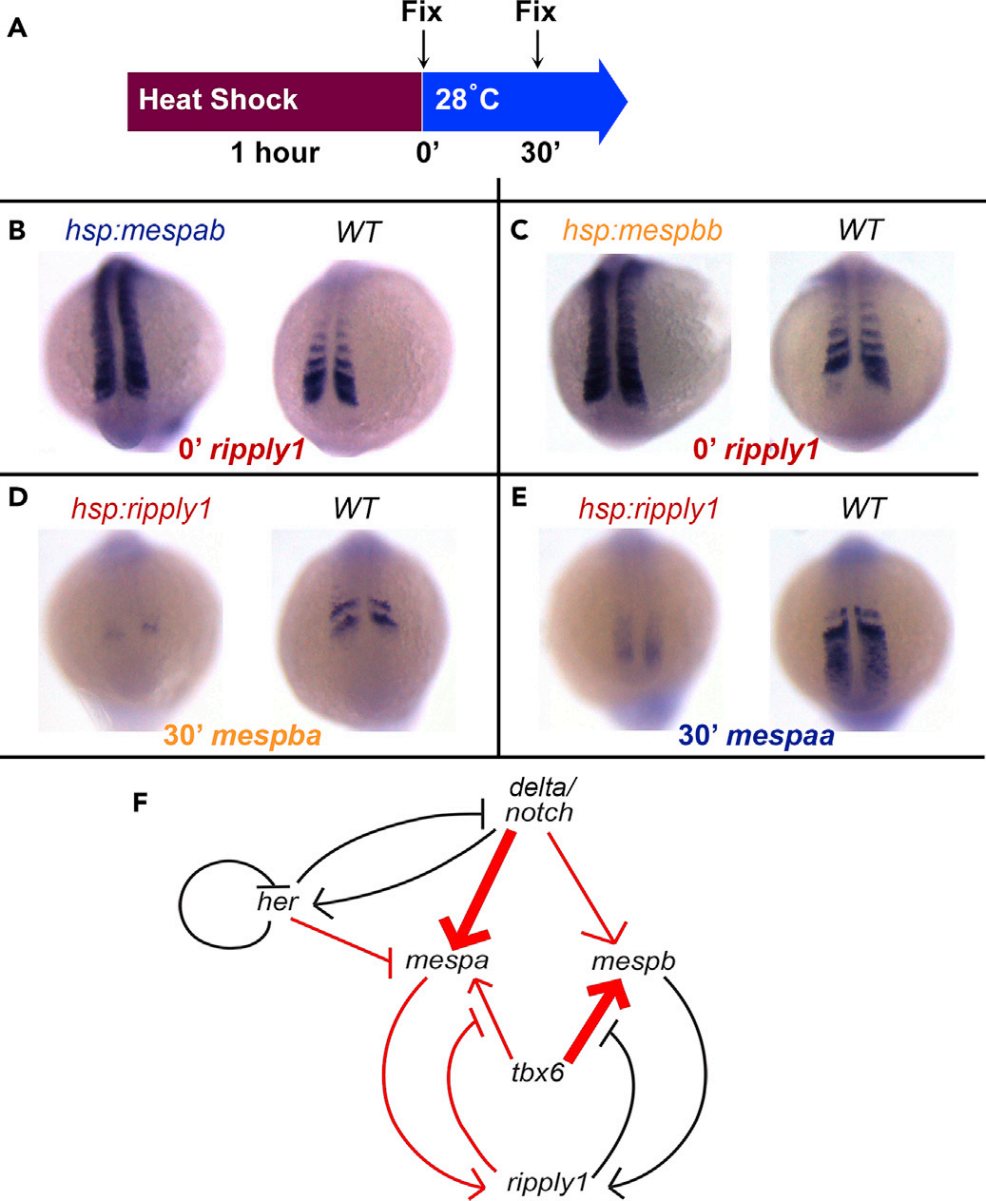
There Is a Negative Feedback Loop between *mespa/mespb* and *ripply1* Genes (A) Embryos from different genetic backgrounds were fixed immediately or 30 min of recovery after 60-min heat shock at 37°C. (B and C) ISH hybridization pictures of *ripply1* gene in (B) *hsp70l:mespab-myc or* (C) *hsp70l:mespbb-myc* embryos after a 1-h heat shock. These experiments were repeated three times, and 104–96 embryos were evaluated for each genotype, respectively. (D and E) ISH hybridization pictures of (D) *mespba* and (E) *mespaa* in *hsp70l:ripply1-myc* embryos after a 1-h heat shock. These experiments were repeated twice and 45–47 embryos were evaluated for all probes. (F) Regulatory network among scoliosis-linked genes. *her* represents both *her1* and *her7*. *delta*/*notch* represents the transcriptional activation of its ligand DeltaC and subsequent pathway activation. Thicker arrows indicate stronger and differential dependence of transcription of each *mesp* gene on two different transcription factors. Red color represents regulatory interactions inferred from time-controlled perturbation experiments in this study. In the computational model, transcription, translation, protein and mRNA degradation, formation of protein complexes, and proteolytic Notch activation are included for the nodes in the network. WT, wild-type.

To assess the regulatory relationship between *mesp* genes and *ripply1*, we used transgenic lines to overexpress *mesp* genes or *ripply1* in a time-controlled manner (Figure 4A). Overexpression of either *mespab* or *mespbb* increased the transcription of *ripply1* immediately after the 60-min heat shock (Figures 4B and 4C), whereas overexpression of *ripply1* decreased transcription of both *mespba* and *mespaa* (Figures 4D and 4E). We combined our data with previous studies of the regulatory connections between *mespba* and *ripply1* (Takahashi et al., 2010, Windner et al., 2015), *ripply1* and *tbx6* (Takahashi et al., 2010, Windner et al., 2015), and *tbx6* and *mespaa* and *mespba* (Sawada et al., 2000, Windner et al., 2015) to construct a gene regulatory network (Figure 4F). The combined information suggested that *mespb* and *ripply1* generate a negative feedback loop: mesp family proteins activate transcription of *ripply1* and Ripply1 reduces the abundance of Tbx6, which is a transcriptional activator of *mesp* genes. We found that transcription of *mespaa* was not affected by the transient overexpression of *mespab* or *mespbb* (Figures 3H and 3I), suggesting that either Tbx6 was not eliminated by transient *mesp* overexpression or that transcription of *mespba* is more sensitive to Tbx6 levels than is *mespaa* transcription. In contrast, transcription of *mespaa* depends more strongly on Notch signaling than does transcription of *mespba* (Figures 3B and 3C). Thus we indicated regulation of the two *mesp* genes with different weights by Notch signaling and Tbx6 in our network of somitogenesis controlled by scoliosis-linked genes with the input of the segmentation clock and its intrinsic feedback as an autoinhibitory loop at the level of *her* (Figure 4F).

The regulatory relationships between the clock (*her*) and Notch (Figure 4F) are based on previous studies, indicating that the main function of Notch signaling is to synchronize oscillations of *her* and *hes* genes in the posterior PSM (Delaune et al., 2012, Mara et al., 2007, Ozbudak and Lewis, 2008, Riedel-Kruse et al., 2007), and our data, showing that *her7* depends on Notch signaling (Figure 3D). However, this dependence is delayed relative to the dependence of *mespaa* expression on Notch signaling (Figure 3B). Thus our regulatory network is consistent with our time-controlled perturbation data in previous Notch gain and loss-of-function experiments in zebrafish and mouse (Feller et al., 2008, Huppert et al., 2005, Ozbudak and Lewis, 2008, Pourquie, 2011). In the regulatory network (Figure 4F), Notch signaling regulates transcription of *mespaa* by an incoherent feedforward loop: Notch activates transcription of *mespaa* independently from the segmentation clock (*her*) and indirectly represses *mespaa* transcription through Notch-mediated stimulation of the *her* family genes.

### Computational Modeling of the Segmentation Network Reproduces Complementary Expression of *her* and *mesp* Family Genes

We built a data-driven mechanistic computational model of the segmentation network (Supplemental Information) to assess whether the regulatory network (Figure 4F) could recapitulate the complementary expression of *mesp* genes with that of *her* and *delta* in somitogenesis (Figure 1B). The model represented the tissue as a two-dimensional hexagonal lattice (4 by 50 cells) and simulated the abundance of *her*, *delta*, and *mesp* mRNAs, proteins, and protein complexes with time-delayed differential equations (see Transparent Methods for details). Biological phenomena are inherently noisy (stochastic) (Ozbudak et al., 2002). To reflect randomness in biochemical reactions of the network (Keskin et al., 2018), we performed pseudo-stochastic simulations of our model as previously described (Ay et al., 2014). Cell-to-cell variability in biochemical reactions was mimicked by assigning different rates for biochemical reactions in each cell (see Methods). This reaction rate variability was kept constant throughout the lifetimes of cells in the PSM (Ay et al., 2014). The simulations utilized previously measured (Ay et al., 2013, Ay et al., 2014, Giudicelli et al., 2007, Hanisch et al., 2013) or physiologically relevant reaction parameters (Table S1). To incorporate the repressive effect of FGF signaling on *mesp* transcription, the model restricts transcription of *mesp* genes only to anterior PSM cells.

**Figure 5:**
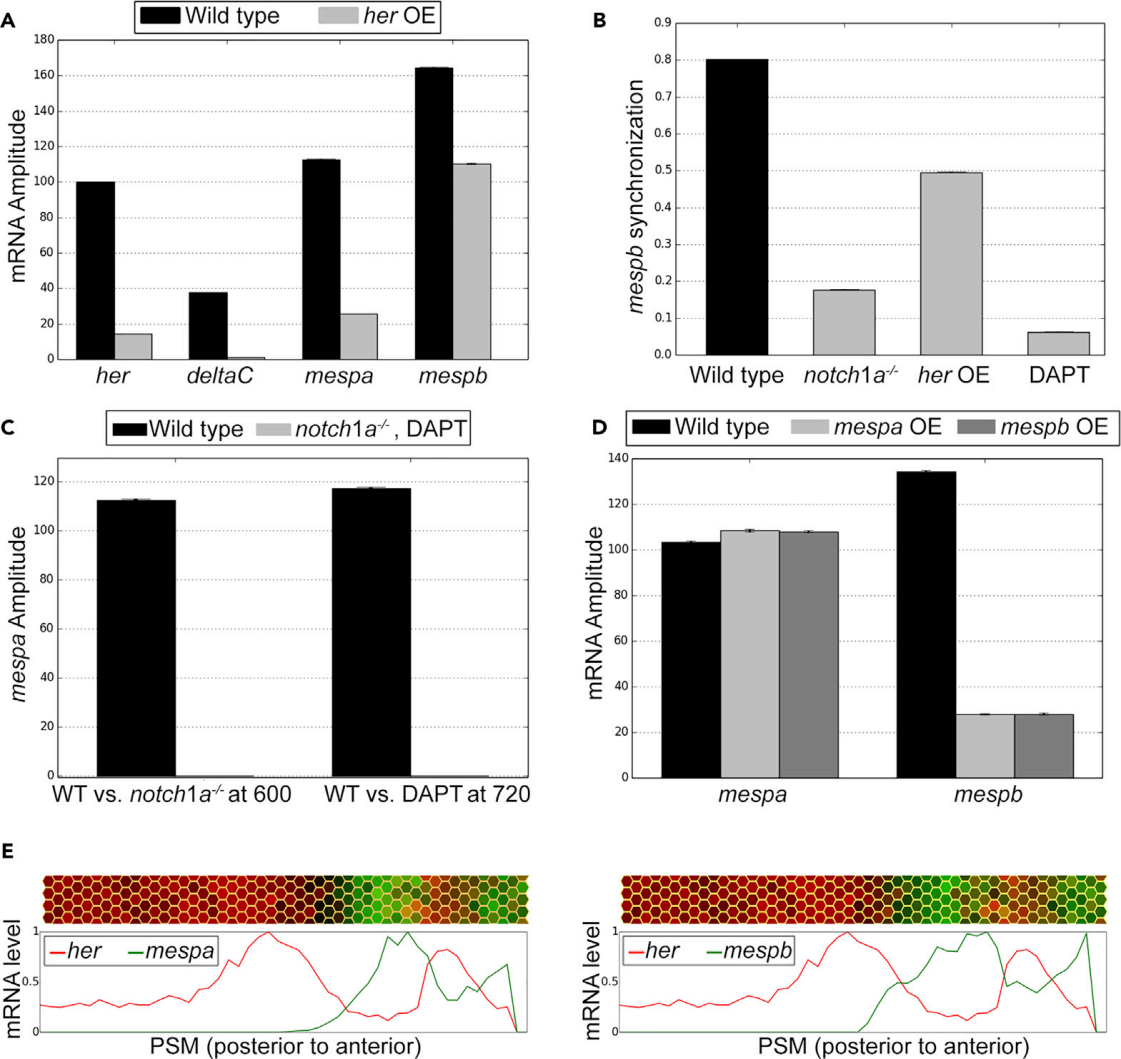
Simulation Results of the Mathematical Model Recapitulate the Experimental Data for Different Conditions (A) Results of simulations showing the effect of *her* overexpression (*her* OE) on *her*, *deltaC*, *mespb*, and *mespa* expression (mRNA Amplitude). (B) Results of simulations showing the effect of the absence of Notch (*notch1a*^*−/−*^ mutant), *her* overexpression, or Notch inhibition with DAPT treatment on synchronized expression of *mespb*. (C) Results of simulations showing the effect of the absence of Notch signaling (*notch1a*^*−/−*^ mutant) or inhibition of Notch signaling with DAPT on *mespa* transcription (mRNA amplitude). (D) Results of simulations showing the effect of *mespab* or *mespbb* overexpression on *mespaa* and *mespb* transcription. To simulate a *notch1a*^*−/−*^ mutant, translation rate of the DeltaC protein (*psd*) was set to zero. To simulate the DAPT condition, *psd* was set to 0 after 600 min. Overexpression of *mespa* and *mespb* genes were simulated by increasing their translation rates *psm*_*a*_ and *psm*_*b*_ from 600 to 660 min. Error bars indicate two standard errors of the mean. (E) Results of simulations of the wild-type (WT) condition showing the pattern of *mesp*a/*b* (green) and *her* (red) transcription. 2D PSM plots with each hexagon representing a “cell”, and line plots are shown on the top and bottom panels, respectively. See also Videos S1, S2, S3, S4, S5, S6, and S7 and Tables S1 and S2.

We first constrained our model parameters by reproducing our earlier results (Ay et al., 2014). These simulations recapitulated previously published experimental data: (1) sustained, synchronized, and stripe-patterned oscillations of the segmentation clock genes in wild-type (Table S1) (Giudicelli et al., 2007); (2) *notch1a*^−/−^ mutant phenotype where clock oscillation period increases (Herrgen et al., 2010), amplitude decreases (Ozbudak and Lewis, 2008), and oscillations desynchronize (Delaune et al., 2012, Horikawa et al., 2006, Jiang et al., 2000, Mara et al., 2007, Ozbudak and Lewis, 2008, Riedel-Kruse et al., 2007) (Table S1); and (3) rapid repression of both *her7* and *deltaC* genes after overexpression of *her7* (Table S1 and Figure 5A) (Giudicelli et al., 2007).

We checked whether our model could reproduce the results of our time-controlled perturbation experiments. Using our model, we found parameter sets that reproduce our experimental data (Videos S1, S2, S3, and S4, and Table S2): (1) repression of *mespa* transcription by clock overexpression (model Figure 5A and Video S1, data Figure 2B), (2) gradual desynchronization of *mespb* transcription by clock overexpression (model Figure 5B and Video S2, data Figure 2C), (3) loss of *mespa* transcription when Notch signaling is blocked (model Figure 5C and Video S3, data Figure 3B), (4) gradual desynchronization of *mespb* transcription when Notch signaling is blocked (model Figures 5B and Video S4, data Figure 3C), and (5) differential dependence of *mespb* and *mespa* expression on overexpression of either gene with *mespb* repressed and *mespa* not affected (model Figure 5D, data Figures 3H and 3I). Note that although we did not impose any spatial restriction on the transcription of *mesp* genes in the anterior PSM, our model readily reproduced complementary transcription of *her* and *mespa/b* genes in wild-type embryos (Figure 5E and Videos S5 and S6). We further tested our model by showing that the striped expression of *mesp* genes are lost in *her* mutants (Video S7) demonstrating that *her* genes drive anteroposterior (A/P) polarized expression of *mesp* genes. However, repression of mespa by *her* genes alone cannot completely accommodate for the complementary expression of *mesp* and *her* genes because (1) *deltaC* is also repressed by *her* genes but is expressed concurrently with *her* genes (Giudicelli et al., 2007) and (2) *mespb* is not repressed by *her* genes (Figure 2C), but similar to *mespa,* it is also expressed in a complementary manner with *her* genes (Cutty et al., 2012, Sawada et al., 2000). Other feedback and feedforward loops in the network (Figure 4F) also contribute to the final dynamic expression patterns, and time-controlled perturbations of these feedback loops also disrupt A/P polarized complementary expression patterns (Figures 2, 3, and 4). Altogether, our results showed that the regulatory network established in this study is sufficient to explain the dynamic expression patterns in wild-type zebrafish embryos and the results of previously published data as well as our time-controlled perturbation experiments.

Video S1. Simulation Reproducing Repression of *mespa* Transcription by Overexpression of *her7* in the PSM, Related to Figure 5 Posterior is to the left, anterior is to the right. Kinematic waves (moving expression stripes) of *her* transcripts are shown in red; dynamic stripes of *mespa* transcripts are shown in green. Zero transcript number is shown in black. Heat shock is applied at snapshot time 29 of the video. *mespa* transcription is quickly repressed. After some time the system recovers and expression patterns return to original state.

Video S2. Simulation Reproducing Gradual Desynchronization of *mespb* Transcription by Overexpression of *her7* in the PSM, Related to Figure 5 Posterior is to the left, anterior is to the right. Kinematic waves (moving expression stripes) of *her* transcripts are shown in red; dynamic stripes of *mespb* transcripts are shown in green. Zero transcript number is shown in black. Heat shock is applied at snapshot time 29 of the video. *mespb* transcription is slowly desynchronized. After some time the system recovers and expression patterns return to original state.

Video S3. Simulation Reproducing Loss of *mespa* Transcription by Inhibition of Notch Signaling in the PSM, Related to Figure 5 Posterior is to the left, anterior is to the right. Kinematic waves (moving expression stripes) of *her* transcripts are shown in red; dynamic stripes of *mespa* transcripts are shown in green. Zero transcript number is shown in black. Notch inhibitor (DAPT) is applied at snapshot time 29 of the video. *mespa* transcription is lost in time. After some time the system recovers and expression patterns return to original state.

Video S4. Simulation Reproducing Gradual Desynchronization of *mespb* Transcription by Inhibition of Notch Signaling in the PSM, Related to Figure 5 Posterior is to the left, anterior is to the right. Kinematic waves (moving expression stripes) of *her* transcripts are shown in red; dynamic stripes of *mespb* transcripts are shown in green. Zero transcript number is shown in black. Notch inhibitor (DAPT) is applied at snapshot time 29 of the video. *mespb* transcription is slowly desynchronized. After some time the system recovers and expression patterns return to original state.

Video S5. Dynamic Expression of her and *mespa* Family Genes in the PSM, Related to Figure 5 Posterior is to the left, anterior is to the right. Kinematic waves (moving expression stripes) of *her* transcripts are shown in red; dynamic stripes of *mespa* transcripts are shown in green. Zero transcript number is shown in black.

Video S6. Dynamic Expression of *mespa* and *mespb* Family Genes in the PSM, Related to Figure 5 Posterior is to the left, anterior is to the right. Dynamic and overlapping stripes of *mespa* and *mespb* transcripts are shown in red and green, respectively. Zero transcript number is shown in black.

Video S7. Simulations Demonstrating Loss of Striped Pattern of mespa Transcription by Mutation of her Family Genes in the PSM, Related to Figure 5 Posterior is to the left, anterior is to the right. *mespa* transcripts are shown in green. Loss-of-function mutations in *her* family genes abolishes striped pattern of *mespa* transcription in the PSM. Striped pattern of *mespb* transcription is also abolished in *her* mutants (video not shown).

## Discussion

Experimental analysis coupled with mathematical modeling is a powerful way to understand complex biological systems. Vertebrate segmentation is an excellent example of such a system, because it involves codependent expression of multiple genes, which is regulated by dynamic signaling pathways. The establishment of rostrocaudal polarity is critical for understanding the etiology and potentially preventing various types of vertebral malformations. Somitogenesis shows the importance of timing in embryonic development. Hence, experiments that generate well-controlled temporal perturbations enable the investigation of questions related to timing using somitogenesis as the model system. By applying this strategy, we identified a high-resolution time course of transcriptional changes (with time window of 30 min to 4 h) and built a time-resolved regulatory network that establishes rostrocaudal polarity of somites in zebrafish. To test the sufficiency of the regulatory network in explaining experimental results, we built a comprehensive computational model. Computational simulations successfully recapitulated experimental results. More importantly, the model was essential to show that the regulatory network that we identified is sufficient to produce complementary expression of *her* and *mesp* genes.

*Mesp* genes are expressed only in the anterior PSM but excluded from the posterior PSM. Their restricted expression to the anterior PSM had been attributed to posterioanterior gradients of FGF or Wnt signaling (Aulehla et al., 2008, Bajard et al., 2014, Delfini et al., 2005, Sawada et al., 2001); however, evidence was missing as to which of these signaling pathways controls the expression domain of *mesp* genes. By performing time-controlled perturbation experiments, we for the first time showed that altering FGF signaling immediately shifted expression domains of *mesp* genes more posteriorly, whereas Wnt signaling only shifted expression after a long delay (Figures 2F–2H). These results extend our previous observation that FGF signaling instructs both the position of the determination front and the anteriorly restricted expression of *mesp* genes, whereas Wnt signaling acts permissively upstream of the FGF signal (Simsek and Ozbudak, 2018). Expression of *mesp* genes in the anterior PSM is restricted to the anterior halves of prospective somites. This rostrocaudally polarized expression of *Mesp* genes has previously been attributed to activation by Notch signaling in the anterior PSM (Oginuma et al., 2010). In mouse, the segmentation clock had been proposed to affect *Mesp* gene expression indirectly through oscillations of Notch signaling. Mutation studies in zebrafish could not clarify whether the Her family clock proteins could regulate *mesp* gene expression more rapidly than Notch signaling and whether this regulation was mediated by activation or repression. Here, we found that the segmentation clock represses the expression of *mespaa*. Furthermore, the expression levels and patterns of both *mespa* and *mespb* have responded faster upon overexpression of Her7 (Figures 2B and 2C) than inhibition of Notch signaling (Figures 3B and 3C). These results suggest that the segmentation clock does not regulate the expression of *mesp* genes via Notch signaling as proposed previously based on steady-state mutant data in mice. Altogether our results indicated that *mesp* genes integrate the spatial information from the FGF gradient (such that *mesp* genes are expressed only in the anterior PSM, but not in the posterior PSM) and the temporal information from the clock (such that *mesp* genes are expressed in rostrocaudally polarized stripes rather than ubiquitously in the anterior PSM). Our results indicated that Notch signaling functions primarily upstream of the segmentation clock in the posterior PSM. Notch signaling regulated the transcription of *mespaa* through an incoherent feedforward loop, and transcription of *mespba* depended more on Tbx6 than on Notch signaling. Cells complete different steps of tissue patterning sequentially by the action of different transcription factors at different spatial locations along the axis.

We here carried out a multidisciplinary approach to map the regulatory network controlling rostrocaudal polarity of somites in the zebrafish model. This blueprint network developed in zebrafish is consistent with many of the phenotypes in mouse (Feller et al., 2008, Huppert et al., 2005, Oginuma et al., 2010, Pourquie, 2011), suggesting that at least the core elements are evolutionarily conserved between fish and mammals. Confirmation of our regulatory network in mammals awaits time-controlled perturbation studies like those we used here. Our model could easily be adapted to mammals at such time that time-controlled perturbation experiments could be performed. We anticipate our interdisciplinary quantitative approach to be adapted to reconstruct regulatory networks governing other dynamic questions in embryonic development.

### Limitations of the Study

The reduction in *mespaa* levels in the *hsp70l:HA-her7* transgenic lines within 30-min heat shock treatment suggested a potential direct regulation of *mespaa* by Her7, whereas the delay in the change in *mespba* and *ripply1* transcripts suggested an indirect regulation of *mespba* and *ripply1* by Her7 (Figures 2B–2D). We have to note that the proof of a direct or indirect transcriptional regulation requires a separate in-depth study of transcriptional binding sites in future.

## Methods

All methods can be found in the accompanying Transparent Methods supplemental file.

## Supplemental Information

Document S1. Transparent Methods, Figure S1, and Tables S1 and S2
