# Supplementary material for: Regulatory Network of the Scoliosis-Associated Genes Establishes Rostrocaudal Patterning of Somites in Zebrafish
Source: iScience. 2019 Jan 21;12:247–59. doi: 10.1016/j.isci.2019.01.021 (PMC6360518; doi:10.1016/j.isci.2019.01.021)
Supplement: Document S1. Transparent Methods, Figure S1, and Tables S1 and S2 [file mmc1.pdf]

ISCI, Volume *12*

Supplemental Information

Regulatory Network of the Scoliosis-Associated Genes Establishes Rostrocaudal Patterning of Somites in Zebrafish

Sevdenur Keskin, M. Fethullah Simsek, Ha T. Vu, Carlton Yang, Stephen H. Devoto, Ahmet Ay, and Ertuğrul M. Özbudak

SUPPLEMENTARY FILES

SUPPLEMENTARY FIGURE AND LEGEND

Figure S1

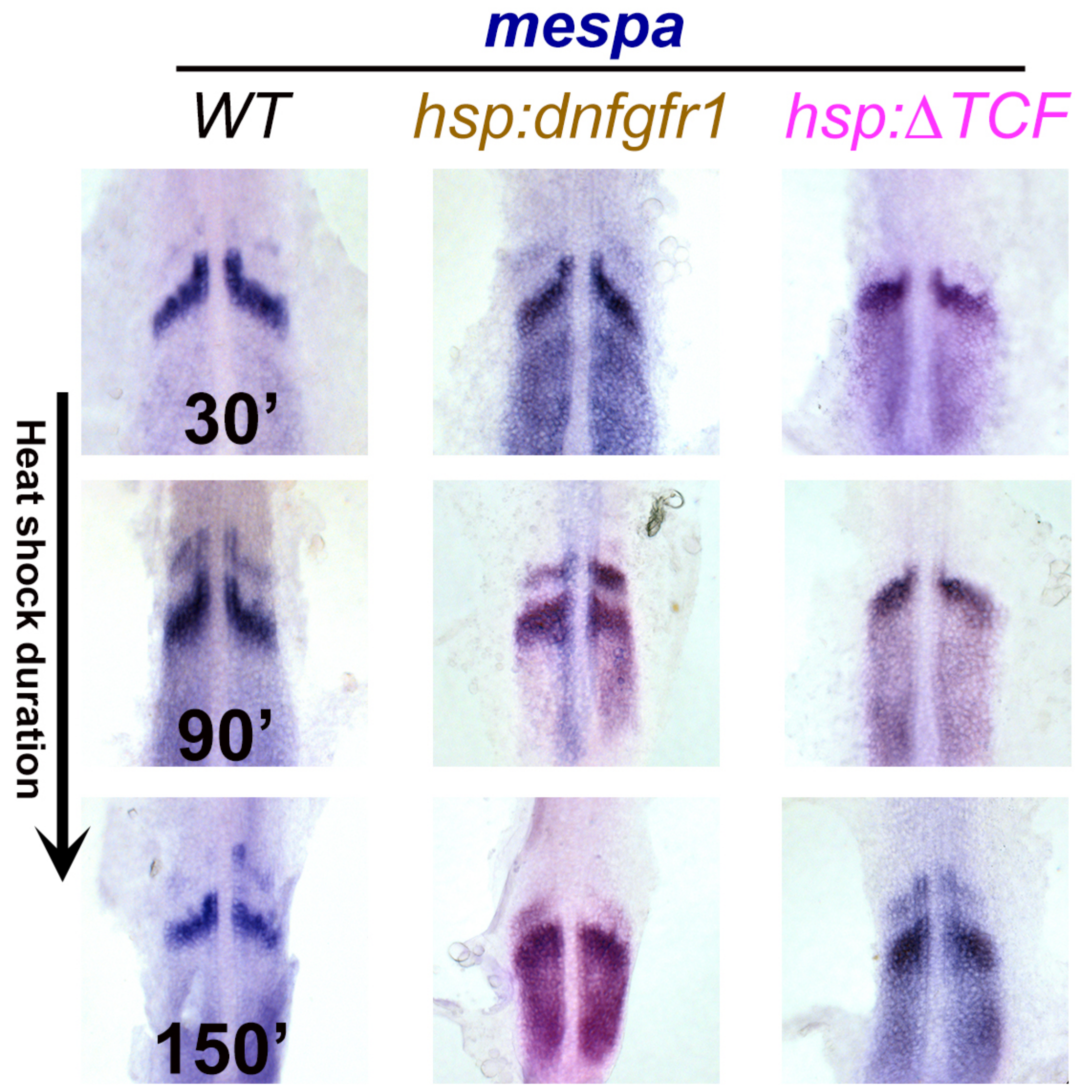

Figure S1. Expression of *mespaa* genes reads out the FGF signaling, Related to Figure 2. Flat mounted ISH images of *mespaa* transcripts at different time points of heat shock of *tg(hsp70l:dnfgfr1a-EGFP)*, *tg(hsp70l:tcf7l1a-GFP)* and wild-type (WT) embryos) at 37 °C. 8 to 22 embryos were flat-mounted for each time points.

SUPPLEMENTARY TABLES

Table S1. Description of Simulation Parameters, Related to Figure 5

Parameter	Description of parameter	Range	Set
msh	her mRNA synthesis rate	[67.2,69.3]	67.4
msm_a	mespa mRNA synthesis rate	[30.1,40.3]	40.2
msm_b	mespb mRNA synthesis rate	[46.6,50.9]	50.1
msd	delta mRNA synthesis	[31.6,33.7]	32.6
mdh	her mRNA degradation	0.5	0.5
mdm_a	mespa mRNA degradation rate	0.1	0.1
mdm_b	mespb mRNA degradation rate	[0.1,0.11]	0.101
mdd	delta mRNA degradation rate	0.5	0.5
psh	her protein synthesis rate	[10.4,10.7]	10.6
psm_a	mespa protein synthesis rate	[34.4,53.4]	43
psm_b	mespb protein synthesis rate	[19.1,21.5]	21.5
psd	delta protein synthesis rate	[26.4,26.6]	26.5
pdh	her protein degradation rate	[0.183,0.185]	0.183
pdm_a	mespa protein degradation rate	[0.155,0.273]	0.157
pdm_b	mespb protein degradation rate	[0.1,0.227]	0.109
pdd	delta protein degradation rate	0.5	0.5
dahh	her-her dimer association rate	0.0003	0.0003
dam_am_a	mespa-mespa dimer association rate	[0.00293,0.00387]	0.00297
dam_am_b	mespa-mespb dimer association rate	[0.0256,0.03]	0.029
dam_bm_b	mespb-mespb dimer association rate	[0.00337,0.00617]	0.00381
ddhh	her-her dimer dissociation rate	[0.194,0.209]	0.202
ddm_am_a	mespa-mespa dimer dissociation rate	[0.00303,0.0252]	0.00497
ddm_am_b	mespa-mespb dimer dissociation rate	[0.214,0.276]	0.27
ddm_bm_b	mespb-mespb dimer dissociation rate	[0.0952,0.3]	0.293
pdhh	her-her dimer degradation rate	[0.17,0.172]	0.171
pdm_am_a	mespa-mespa dimer degradation rate	[0.179,0.222]	0.202
pdm_am_b	mespa-mespb dimer degradation rate	[0.1,0.109]	0.1
pdm_bm_b	mespb-mespb dimer degradation rate	[0.438,0.489]	0.439
delaymh	her mRNA synthesis delay rate	[7,7.01]	7.01
$delaymm_a$	mespa mRNA synthesis delay rate	15	15
$delaymm_b$	mespb mRNA synthesis delay rate	[14.6,15]	14.8
delaymd	delta mRNA synthesis delay rate	[8.99,9.05]	9
delayph	her protein synthesis delay rate	1.14	1.14
$delaypm_a$	mespa protein synthesis delay rate	[0.4,0.524]	0.407
$delaypm_b$	mespb protein synthesis delay rate	[0.4,0.47]	0.405
delaypd	delta protein synthesis delay rate	12.6	12.6

$delta_M$	delay for indirect mesp-ripply-tbx6 feedback	[42.7,43.1]	42.7
critphh	critical binding rate of her-her dimer	[390,406]	394
critipd	critical binding rate of delta protein	[603,651]	651
$critpm_am_a$	cirtical binding rate of mespa-mespa dimer	[1679,1986]	1984
$critpm_bm_b$	critical binding rate of mespb-mespb dimer	[500,659]	501
oeher	her over expression rate	[27.8,31]	29.7
oemespa	mespa over expression rate	[30.9,96.3]	53.5
oemespb	mespb over expression rate	[51.9,84.3]	66.7

Table S2. Description of Simulation Conditions, Related to Figure 5

Genetic Background	Tested Condition	Check Time
Wildtype:		
	Her mRNA oscillation period is ~30 minutes.	100 – 300
	Her mRNA expression shows sustained oscillation.	100 – 300
	Her mRNA period increases from posterior end of the PSM to the anterior end of the PSM.	600 – 900
	Her mRNA oscillations are synchronized between neighboring cells.	600 – 630
	Her and *mespa* mRNAs show complementary pattern in the anterior PSM.	600 – 630
***Notch1a*$^{-/-}$ *mutant:* DeltaC protein synthesis is set to 0.**		
	Her mRNA period increases 7%-20% in *notch1a*$^{-/-}$ mutant.	100 – 300
	Her mRNA amplitude decreases 15%-70% in *notch1a*$^{-/-}$ mutant.	600 – 630
	Her mRNA oscillations are desynchronized in *notch1a*$^{-/-}$ mutant.	600 – 630
	Mespa mRNA amplitude decreases more than 70% in *notch1a*$^{-/-}$ mutant.	600 – 630
	Mespb mRNA oscillations are desynchronized in *notch1a*$^{-/-}$ mutant.	600 – 630
Her overexpression: Her protein synthesis is increased for 30 min starting after 600 min.		
	Her mRNA amplitude decreases more than 70% in 30 minutes after *her* overexpression.	630 – 660
	DeltaC mRNA amplitude decreases more than 70% in 30 minutes after *her* overexpression.	630 – 660
	Mespa mRNA amplitude decreases more than 70% in 30 minutes after *her* overexpression.	630 – 660
	Mespb mRNA oscillations are desynchronized in 1.5 hours after *her* overexpression.	690 – 720
Notch signaling disruption by DAPT: DeltaC protein synthesis is set to 0 after 600 min.		
	Her mRNA oscillations are desynchronized in 4 hours after DAPT treatment.	840 – 870
	Her mRNA amplitude decreases 15%-70% in 4 hours after DAPT treatment.	840 – 870
	Mespa mRNA amplitude decreases more than 70% in 2 hours after DAPT treatment.	720 – 750
	Mespb mRNA oscillations are desynchronized in 4 hours after DAPT treatment.	840 – 870
Mespa overexpression: Mespa protein synthesis is increased for 60 min starting after 600 min.		
	Mespb mRNA amplitude decreases more than 70% in 1 hour after *mespa* overexpression.	660 – 690
Mespb overexpression: Mespb protein synthesis is increased for 60 min starting after 600 min.		
	Mespb mRNA amplitude decreases more than 70% in 1 hour after *mespb* overexpression.	660 – 690

TRANSPARENT METHODS

CONTACT FOR REAGENT AND RESOURCE SHARING

Further information and requests for reagents may be directed to the Lead Contact Ertugrul Ozbudak (Ertugrul.Ozbudak@cchmc.org).

EXPERIMENTAL MODEL AND SUBJECT DETAILS

Fish stocks

All the fish experiments were performed under the ethical guidelines of Albert Einstein College of Medicine and Cincinnati Children's Hospital Medical Center, and animal protocols were reviewed and approved by the respective Institutional Animal Care and Use Committees (Protocol # 20150704 and Protocol # 2017-0048). Fish were kept on a 14-10 light/dark cycle at the Zebrafish Core Facility, maintained at 28.5°C. Transgenic lines *hsp70:HA-her7* (Giudicelli et al., 2007), *hsp70l:mespab-myc, hsp70l:mespbb-myc, hsp70l:ripply1-myc* (Windner et al., 2015) *hsp70l:dnfgfr1a-EGFP* (Lee et al., 2005) and *hsp70l:tcf7l1a-GFP* (Lewis et al., 2004) were used during this study.

METHOD DETAILS

Heat-shock procedures

We used heat-shock inducible promoters to perform time-controlled perturbation experiments throughout this study. This approach induces transgenes very rapidly (Giudicelli et al., 2007) as compared to alternative Tet-on inducible system (Watanabe et al., 2007; Wehner et al., 2015). Transgenic heterozygous fishes were crossed to wild-type fish to obtain transgenic and control embryos with equal proportions. Embryos were kept at a temperature range of 23-28°C until the desired stage for heat-shock. They were then transferred to pre-warmed E3 medium in a 37°C incubator for the desired length of time, then fixed immediately in ice-cold 4% paraformaldehyde or returned to 28°C for further development and fixation at a late recovery time point (Giudicelli et al., 2007). Temporal loss of function of Notch signaling was accomplished by exposing embryos to 100 μM of the γ-secretase inhibitor, *N*-[N-(3,5-difluorophenacetyl)-L-alanyl]-S-phenylgly cine t-butyl ester (DAPT) in DMSO (Ozbudak and Lewis, 2008). Control embryos were exposed to DMSO.

In situ hybridization

In situ hybridization was performed according to standard protocols. Digoxigenin-labelled RNA probes were as previously described for *her1* (Takke and Campos-Ortega, 1999), *her7* (Henry et al., 2002; Oates and Ho, 2002), *deltaC* (Jiang et al., 2000) and *cb1045* (*xirp2a* – Zebrafish Information Network) (Riedel-Kruse et al., 2007). Probes for *mespaa*, *mespba*, and *ripply1* are generated using the nucleotides in between 1-887, 1-801, and 86-785, respectively. We validated the genotype of the selected embryos by PCR by using the following primers: mespabUprv: TCAACATTGGCATTTTCAGG, mespabF_NOT1: gatcGCGGCCGC GCATTCACTCAAGCTCCAGA, mespbbR_ECOR1: gatcGAATTCCAGTGGACGCCTTTGTTGTA, mespbbF_NOT1: gatcGCGGCCGCTAGCGGTGGTCTGGACAGG, shHsp70l_BbvCIfw: gatcCCTCAGCCACACAACCGCACATTTTTC and ripply1rv: CCTCGACGTCACTTTCATCA.

QUANTIFICATION AND STATISTICAL ANALYSIS

Mathematical model

We developed a delayed differential equation model (DDE) with 12 equations (see below) and 44 parameters (Table S1). Each equation in our DDE model represents the rate of change of a model state (mRNA, protein, or protein complex); each model parameter represents the rate of the corresponding reaction that influences the concentrations of the model states. Biological reaction terms describe the synthesis and degradation of mRNAs and proteins, as well as dimer association, dissociation, and degradation.

The genes included in the model are *her, deltaC, mespa* and *mespb*. Following Lewis (2003) (Lewis, 2003), we represented *her1* and *her7* genes as one *her* gene. In our model, Her protein forms Her-Her homodimer, and represses transcription of *her, deltaC*, and *mespa* genes (Figure 5E). Mesp proteins form dimers to repress transcription of *mespb* gene. DeltaC triggers the proteolytic cleavage of the Notch protein intracellular domain (NICD). NICD translocates into the nucleus and activates the transcription of *her, mespa*, and *mespb.* The transcriptional repressors Her-Her, Mespa-Mespa, and Mespb-Mespb compete with the NICD protein for binding to the DNA regulatory region to repress transcription of *her*, *mespa* and *mespb* genes (Ozbudak and Lewis, 2008). To simplify the model, we followed earlier work (Ay et al., 2014; Lewis, 2003) and did not explicitly write an equation representing the production of NICD. Instead, we represented NICD levels in each cell as a function of the DeltaC protein levels in all neighboring cells.

The variables: mh, md, mm_a and mm_b represent the number of mRNA molecules of *her*, *deltaC*, *mespa* and *mespb* respectively; ph, pd, pm_a and pm_b represent the number of protein molecules of Her, DeltaC, Mespa and Mespb respectively; and phh, pm_am_a, pm_bm_b and pm_am_b represent the number of molecules of Her-Her, Mespa-Mespa, Mespb-Mespb and Mespa-Mespb dimers, respectively. mRNA synthesis rates are denoted as msh, msd, msm_a and msm_b for *her*, *deltaC*, *mespa* and *mespb* genes, respectively. mRNA degradation rates are denoted as mdh, mdd, mdm_a and mdm_b for *her*, *deltaC*, *mespa* and *mespb* mRNAs, respectively. Protein synthesis rates are denoted as psh, psd, psm_a and psm_b for Her, DeltaC, Mespa and Mespb proteins, respectively. Degradation rates for Her, DeltaC, Mespa and Mespb proteins are denoted as pdh, pdd, pdm_a and pdm_b, respectively. Dimer association, dissociation, and degradation rates for Her-Her are represented by $dahh$, $ddhh$ and $pdhh$, respectively. Dimer association, dissociation, and degradation rates for Mespa-Mespa, Mespb-Mespb and Mespa-Mespb are represented by dam_am_a, ddm_am_a, pdm_am_a, dam_bm_b, ddm_bm_b, pdm_bm_b, dam_am_b, ddm_am_b, and pdm_am_b, respectively. DNA-binding dissociation rates are $critphh$, $critpd$, $critpm_am_a$, and $critpm_bm_b$ for Her-Her, Notch (NICD), Mespa-Mespa, and Mespb-Mespb, respectively. Transcriptional time delays of *her*, *deltaC*, *mespa*, and *mespb* mRNAs include the transcription, splicing, and nuclear-to-cytoplasmic transport, and these delays are represented by $delaymh$, $delaymd$, $delaym_a$, and $delaym_b$, respectively. The translational time delays of Her, Mespa and Mespb proteins include translation and nuclear import of these repressor proteins, and these delays are represented by $delayph$, $delayp_a$, and $delayp_b$, respectively. The translational time delay of DeltaC protein includes translation and localization of DeltaC protein at the membrane, interaction of Delta-Notch proteins, and production and localization of NICD at the nucleus, and is expressed as *delaypd.* Likewise, we defined the time-delay δ_M , to represent the total time-delay in the indirect regulatory interactions between Mesp, Ripply1, and Tbx6 proteins (Windner et al., 2015). We represent the kth cell as c_k and time as t.

Delay Differential Equation Model:

A. mRNA Levels

A.1. her mRNA Levels

$$\frac{\partial mh(c_k,t)}{\partial t} = msh \frac{1+\frac{1}{6}\sum_{c_n \in N} \frac{pd(c_n,t-delaymh)}{critpd}}{1+\frac{1}{6}\sum_{c_n \in N} \frac{pd(c_n,t-delaymh)}{critpd} + [\frac{phh(c_k,t-delaymh)}{critphh}]^2} - mdh \cdot mh(c_k,t)$$

A.2. deltaC mRNA Levels

$$\frac{\partial md(c_k,t)}{\partial t} = msd \frac{1}{1+[\frac{phh(c_k,t-delaymd)}{critphh}]^2} - mdd \cdot md(c_k,t)$$

A.3. mespa mRNA Levels

$$\frac{\partial mm_a(c_k,t)}{\partial t} = msm_a \frac{\frac{1}{6}\sum_{c_n \in N} \frac{pd(c_n,t-delaymm_a)}{critpd}}{1+\frac{1}{6}\sum_{c_n \in N} \frac{pd(c_n,t-delaymm_a)}{critpd} + [\frac{phh(c_k,t-delaymm_a)}{critphh}]^2} - mdm_a \cdot m_a(c_k,t)$$

A.4. mespb mRNA Levels

$$\frac{\partial mm_b(c_k,t)}{\partial t} = msm_b \frac{1+\frac{1}{6}\sum_{c_n \in N} \frac{pd(c_n,t-delaymm_b)}{critpd}}{\left[\begin{array}{l} 1+\frac{1}{6}\sum_{c_n \in N} \frac{pd(c_n,t-delaymm_b)}{critpd} + [\frac{pm_a m_a(c_k,t-delaymm_b-\delta_M)}{critpm_a m_a}]^2 \\ +[\frac{pm_b m_b(c_k,t-delaymm_b-\delta_M)}{critpm_b m_b}]^2 \end{array}\right]} - mdm_b \cdot m_b(c_k,t)$$

where N represents all the neighbors of the kth cell (c_k)

B. Monomer Protein Levels

B.1. Her Monomer Protein Levels

$$\frac{\partial ph(c_k,t)}{\partial t} = psh \cdot mh(c_k,t-delayph) - pdh \cdot ph(c_k,t) + 2ddhh \cdot phh(c_k,t) - 2dahh \cdot ph(c_k,t) \cdot ph(c_k,t)$$

B.2. Delta Monomer Protein Levels

$$\frac{\partial pd(c_k,t)}{\partial t} = psd \cdot md(c_k,t-delaypd) - pdd \cdot pd(c_k,t)$$

B.3. Mespa Monomer Protein Levels

$$\frac{\partial pm_a(c_k,t)}{\partial t} = psm_a \cdot mm_a(c_k,t-delaypm_a) - pdm_a \cdot pm_a(c_k,t) + 2ddm_am_a \cdot pm_am_a(c_k,t) - 2dam_am_a \cdot pm_a(c_k,t) \cdot pm_a(c_k,t) + ddm_am_b \cdot pm_am_b(c_k,t) - dam_am_b \cdot pm_a(c_k,t) \cdot pm_b(c_k,t)$$

B.4. Mespb Monomer Protein Levels

$$\frac{\partial pm_b(c_k,t)}{\partial t} = psm_b \cdot mm_b(c_k,t-delaypm_b) - pdm_b \cdot pm_b(c_k,t) + 2ddm_bm_b \cdot pm_bm_b(c_k,t) - 2dam_bm_b \cdot pm_b(c_k,t) \cdot pm_b(c_k,t) + ddm_am_b \cdot pm_am_b(c_k,t) - dam_am_b \cdot pm_a(c_k,t) \cdot pm_b(c_k,t)$$

C. Dimer Protein Levels

C.1. Her-Her Dimer Protein Levels

$$\frac{\partial phh(c_k,t)}{\partial t} = -ddhh \cdot phh(c_k,t) + dahh \cdot ph(c_k,t) \cdot ph(c_k,t) - pdhh \cdot phh$$

C.2. Mespa-Mespa Dimer Protein Levels

$$\frac{\partial pm_a m_a(c_k,t)}{\partial t} = -ddm_a m_a \cdot pm_a m_a(c_k,t) + dam_a m_a \cdot pm_a(c_k,t) \cdot pm_a(c_k,t) - pdm_a m_a \cdot pm_a m_a$$

C.3. Mespb-Mespb Dimer Protein Levels

$$\frac{\partial pm_b m_b(c_k,t)}{\partial t} = -ddm_b m_b \cdot pm_b m_b(c_k,t) + dam_b m_b \cdot pm_b(c_k,t) \cdot pm_b(c_k,t) - pdm_b m_b \cdot pm_b m_b$$

C.4. Mespa-Mespb Dimer Protein Levels

$$\frac{\partial pm_a m_b(c_k,t)}{\partial t} = -ddm_a m_b \cdot pm_a m_b(c_k,t) + dam_a m_b \cdot pm_a(c_k,t) \cdot pm_b(c_k,t) - pdm_a m_b \cdot pm_a m_b$$

Spatial modeling

A two-dimensional hexagonal grid of 4 × 50 cells was used to represent the PSM tissue in our simulations. The right- and left-most cells in each column were connected artificially, such that each cell has six neighbors, excluding the cells located in the most posterior and anterior columns that have only four neighbors. The model was simulated for 930 min in total. In the first 300 min, we simulated only 4 × 10 cells forming the posterior PSM. Then, we grew the posterior PSM tissue for 240 min until the PSM was full by adding a column of 4 cells every six minutes. After the PSM was full, we added a column of 4 cells at the posterior end, and removed an older column of cells at the anterior end to keep a fixed PSM size. We set the Her and DeltaC translational time delays within a biologically relevant range in the posterior PSM (first 10 columns of cells); this rate was linearly interpolated at all intermediate (middle 20 columns of cells) and anterior PSM locations (last 20 columns of cells). The largest translational time delays at the anterior PSM for Her and DeltaC proteins were set to 3.9 fold of posterior PSM (Ay et al., 2014). Similarly, the translational time delays of Mespa and Mespb proteins were increased 2.1 fold from mid-PSM to anterior PSM. The model implicitly implements the input of FGF signaling on *mesp* transcription, by restricting the transcription zone of *mesp* genes only to anterior PSM in simulations.

Pseudo-stochastic numerical simulation

We carried out pseudo-stochastic simulations of our delay differential equation model to reproduce randomness in the regulatory network. Biochemical reaction rates (parameters in the model) were allowed to change up to 16% between cells to generate the inherent stochasticity in this biological system. The variations in reaction rates were formed during the creation of the cells and kept constant throughout the lifetime of the cells in the PSM. The perturbed system of DDEs was solved numerically using Euler's method. Euler's method increments the time by the

chosen step size (0.01 min), and updates mRNA and mono and dimer protein levels after each iteration using the rates of change specified by the DDEs. To simulate a *notch1a*$^{-/-}$ mutant, we set the translation rate of the DeltaC protein (*psd*) to zero. To simulate the DAPT condition, we set *psd* to 0 after 600 min. Overexpression of *her* gene was modeled by increasing its translation rate *psh* from 600 to 630 min. Similarly, we modeled overexpression of *mespa* and *mespb* genes by increasing their translation rates psm_a and psm_b from 600 to 660 min.

Parameter estimation

Delays in transcription and translation and degradation rates of mRNA and protein have been measured experimentally (Ay et al., 2014; Ay et al., 2013; Giudicelli et al., 2007; Hanisch et al., 2013). However, some of the reaction rates have not been measured due to technical limitations. We used parameter estimation to identify biologically relevant reaction rates (model parameters) that could reproduce the experimental observations.

Parameter search was performed using the stochastic ranking evolutionary strategy (SRES) algorithm (Runarsson and Yao, 2000), which looked for suitable parameter sets fit to experimental conditions (Table S2). The SRES algorithm performs better than other parameter estimation algorithms in large-scale non-linear biological systems (Fakhouri et al., 2010; Fomekong-Nanfack et al., 2007; Moles et al., 2003). We used the ranges provided in Ay *et al.* (2014) for the parameters that represent the same functionality as the model from (Ay et al., 2014). New parameters were given freedom within biologically realistic limits. To produce biologically feasible parameter ranges (Table S1), parameter ranges were refined a few times based on the results of initial parameter searches.

Posterior PSM

The DDE model was simulated for 300 min in 40 (4 rows of 10 cells) posterior PSM cells to obtain *her* mRNA expression levels. The period was calculated as the time difference between the last two peaks of *her* mRNA oscillations.

Whole PSM

The PSM reached its full size of 200 cells in 540 min in our simulations. To calculate the amplitude and synchrony of segmentation network genes, we took 1 snapshot for overexpression experiments, and 5 snapshots for *notch1a*$^{-/-}$ mutant and DAPT treatment over 30 min of simulation.

The amplitudes for overexpression embryos were calculated as the change between the average of top ten and bottom ten corresponding gene expression levels. The amplitudes for *notch1a*$^{-/-}$ mutant and DAPT embryos were calculated as the mean of the five measured amplitudes found using five snapshots. The synchronization scores for overexpression transgenics were measured by finding average of the three Pearson correlation coefficients measured between each row of cells and the first row of cells. The synchronization scores for *wildtype*, *notch1a*$^{-/-}$, and DAPT embryos were calculated as the mean of the five calculated synchronization scores found using five snapshots. The complementarity between *her* and *mespa/mespb* genes were calculated as the average of the fifteen (five snapshots, four rows) Pearson correlation coefficients between *her* and *mespa/mespb* gene expression levels.

Coding

The model and related analysis are implemented in C++ and Python. C++ is used for the model's numerical simulation and parameter search, because of its speed. Python is used due to its user-friendly and superior data processing and plotting libraries. Our code can perform a 930

min simulation of 200 PSM cells in less than 1 min on an iMAC running MAC OSX 10.12.4 with 3.1 GHz Intel Core i7 and 16 GB of RAM. A parallel version of our code is written using the Message Passing Interface (MPI) for time-intensive parameter searches. Each SRES parameter estimation run with a population size of 20, 3 parents, and 2,000 generations took approximately 48 hours using 24 processors on a computer cluster of 19 nodes, 248 processors, and 24 gigabytes of RAM per node.

DATA AND SOFTWARE AVAILABILITY

The C++ and Python codes will be made available upon request.

REFERENCES

Ay, A., Holland, J., Sperlea, A., Devakanmalai, G.S., Knierer, S., Sangervasi, S., Stevenson, A., and Ozbudak, E.M. (2014). Spatial gradients of protein-level time delays set the pace of the traveling segmentation clock waves. Development *141*, 4158-4167.

Ay, A., Knierer, S., Sperlea, A., Holland, J., and Özbudak, E.M. (2013). Short-lived Her Proteins Drive Robust Synchronized Oscillations in the Zebrafish Segmentation Clock. Development *140*, 3244-3253.

Fakhouri, W.D., Ay, A., Sayal, R., Dresch, J., Dayringer, E., and Arnosti, D.N. (2010). Deciphering a transcriptional regulatory code: modeling short-range repression in the Drosophila embryo. Molecular Systems Biology *6*.

Fomekong-Nanfack, Y., Kaandorp, J.A., and Blom, J. (2007). Efficient parameter estimation for spatio-temporal models of pattern formation: case study of Drosophila melanogaster. Bioinformatics *23*, 3356-3363.

Giudicelli, F., Ozbudak, E.M., Wright, G.J., and Lewis, J. (2007). Setting the Tempo in Development: An Investigation of the Zebrafish Somite Clock Mechanism. PLoS Biol *5*, e150.

Hanisch, A., Holder, M.V., Choorapoikayil, S., Gajewski, M., Ozbudak, E.M., and Lewis, J. (2013). The elongation rate of RNA Polymerase II in the zebrafish and its significance in the somite segmentation clock. Development *140*, 444-453.

Henry, C.A., Urban, M.K., Dill, K.K., Merlie, J.P., Page, M.F., Kimmel, C.B., and Amacher, S.L. (2002). Two linked hairy/Enhancer of split-related zebrafish genes, her1 and her7, function together to refine alternating somite boundaries. Development *129*, 3693-3704.

Jiang, Y.J., Aerne, B.L., Smithers, L., Haddon, C., Ish-Horowicz, D., and Lewis, J. (2000). Notch signalling and the synchronization of the somite segmentation clock. Nature *408*, 475-479.

Lee, Y., Grill, S., Sanchez, A., Murphy-Ryan, M., and Poss, K.D. (2005). Fgf signaling instructs position-dependent growth rate during zebrafish fin regeneration. Development *132*, 5173-5183.

Lewis, J. (2003). Autoinhibition with transcriptional delay: A simple mechanism for the zebrafish somitogenesis oscillator. Current Biology *13*, 1398-1408.

Lewis, J.L., Bonner, J., Modrell, M., Ragland, J.W., Moon, R.T., Dorsky, R.I., and Raible, D.W. (2004). Reiterated Wnt signaling during zebrafish neural crest development. Development *131*, 1299-1308.

Moles, C.G., Mendes, P., and Banga, J.R. (2003). Parameter estimation in biochemical pathways: A comparison of global optimization methods. Genome Research *13*, 2467-2474.

Oates, A.C., and Ho, R.K. (2002). Hairy/E(spl)-related (Her) genes are central components of the segmentation oscillator and display redundancy with the Delta/Notch signaling pathway in the formation of anterior segmental boundaries in the zebrafish. Development *129*, 2929-2946.

Ozbudak, E.M., and Lewis, J. (2008). Notch signalling synchronizes the zebrafish segmentation clock but is not needed to create somite boundaries. PLoS genetics *4*, e15.
Riedel-Kruse, I.H., Muller, C., and Oates, A.C. (2007). Synchrony dynamics during initiation, failure, and rescue of the segmentation clock. Science *317*, 1911-1915.
Runarsson, T.P., and Yao, X. (2000). Stochastic ranking for constrained evolutionary optimization. Ieee T Evolut Comput *4*, 284-294.
Takke, C., and Campos-Ortega, J.A. (1999). her1, a zebrafish pair-rule like gene, acts downstream of notch signalling to control somite development. Development *126*, 3005-3014.
Watanabe, T., Saito, D., Tanabe, K., Suetsugu, R., Nakaya, Y., Nakagawa, S., and Takahashi, Y. (2007). Tet-on inducible system combined with in ovo electroporation dissects multiple roles of genes in somitogenesis of chicken embryos. Dev Biol *305*, 625-636.
Wehner, D., Jahn, C., and Weidinger, G. (2015). Use of the TetON System to Study Molecular Mechanisms of Zebrafish Regeneration. Jove-J Vis Exp.
Windner, S.E., Doris, R.A., Ferguson, C.M., Nelson, A.C., Valentin, G., Tan, H., Oates, A.C., Wardle, F.C., and Devoto, S.H. (2015). Tbx6, Mesp-b and Ripply1 regulate the onset of skeletal myogenesis in zebrafish. Development *142*, 1159-1168.
